# Sodium Valproate-Induced Gingival Enlargement in a Pediatric Patient: A Case Report With Surgical Intervention

**DOI:** 10.7759/cureus.86519

**Published:** 2025-06-22

**Authors:** Nezy Varghese, R Rajesh, Darsana Krishnan, Sreelakshmi Vasu

**Affiliations:** 1 Pediatric and Preventive Dentistry, Indira Gandhi Institute of Dental Sciences, Nellikuzhi, IND

**Keywords:** gingival overgrowth, gingivectomy, oral hygiene, plaque, sodium valproate

## Abstract

Gingival enlargement occurs secondary to the usage of anti-epileptics. The severity of overgrowth varies among different classes of drugs, with phenytoin having the highest incidence of reported cases, whereas cases associated with sodium valproate are minimal. Factors like drug dose, genetic factors, plaque, gingival inflammation, and periodontal health influence the degree of overgrowth. This case report presents one of the first surgically managed cases of generalized gingival enlargement in a pediatric patient due to chronic usage of sodium valproate.

## Introduction

Drug-induced gingival enlargement refers to an abnormal growth of the gingiva secondary to the use of systemic medication and is classified by the 2017 World Workshop as a form of dental plaque-induced gingival disease modified by medications [1]. Currently, three categories of medications (anticonvulsants, calcium channel blockers, and immunosuppressants) are associated with gingival enlargement [2]. Only a few cases with chronic use of sodium valproate in children and adults have been documented and the percentage of overgrowth due to valproate is lesser than phenytoin [3,4].

This case report describes a pediatric patient with generalized gingival enlargement, managed surgically through gingivectomy and gingivoplasty, in conjunction with strict oral hygiene measures.

## Case presentation

A twelve-year-old female patient presented to the Department of Pediatric Dentistry with a two-year history of generalized gingival swelling and bleeding while brushing. No pain was reported. The patient was esthetically concerned and had difficulty maintaining oral hygiene. Medical history revealed epileptic episodes for which the patient was under sodium valproate medication. The first episode of epilepsy occurred at six years of age, for which the patient was prescribed the tablet Valparin 200 mg twice daily; the medication was discontinued after three years. The second episode occurred at 10 years of age, following which the patient was prescribed the tablet Valparin 300 mg twice daily, after which gum swelling was observed.

**Figure 1:**
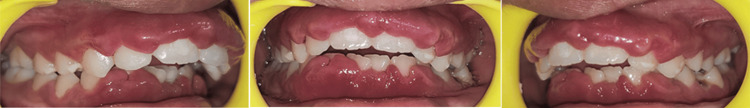
Intraoral photographs showing sodium valproate-induced generalized gingival enlargement

Intraoral examination showed the presence of plaque and dental calculus on the upper and lower teeth along with generalized gingival enlargement (Figure 1). A gingivectomy followed by oral hygiene prophylaxis was planned as part of the treatment. Although the gingival enlargement will recur, gingivectomy was selected as a treatment option as it would improve oral hygiene and esthetic profile. The drug was not altered as advised by the concerned physician. The consent form was obtained. Patients routine blood checkup was performed, which was within normal limits (Table 1).

**Table 1:** Complete blood count report

Investigation (blood sample)	Result	Interpretation	Reference value	Unit
Hemoglobin	13	Normal	13-17	g/dL
Total RBC count	5	Normal	4.50-5.50	Mill/cumm
PCV	45	Normal	45-50	%
MCV	96	Normal	83-101	fL
MCH	30	Normal	27-32	pg
MCHC	33	Normal	32.50-34.50	g/dL
Total WBC count	10000	Normal	4000-11000	Cumm
Bleeding time	6	Normal	2-7	minutes
Clotting time	8	Normal	4-11	minutes

**Figure 2:**
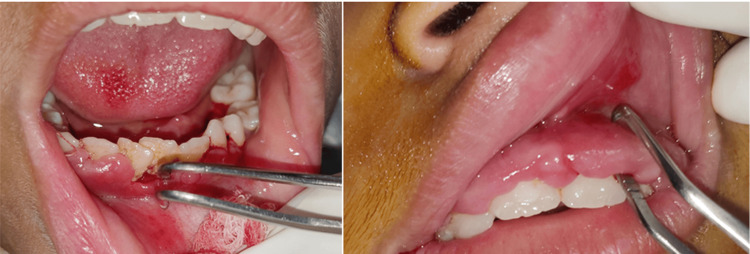
Pocket depth marked using a pocket marker, with bleeding points visible

**Figure 3:**
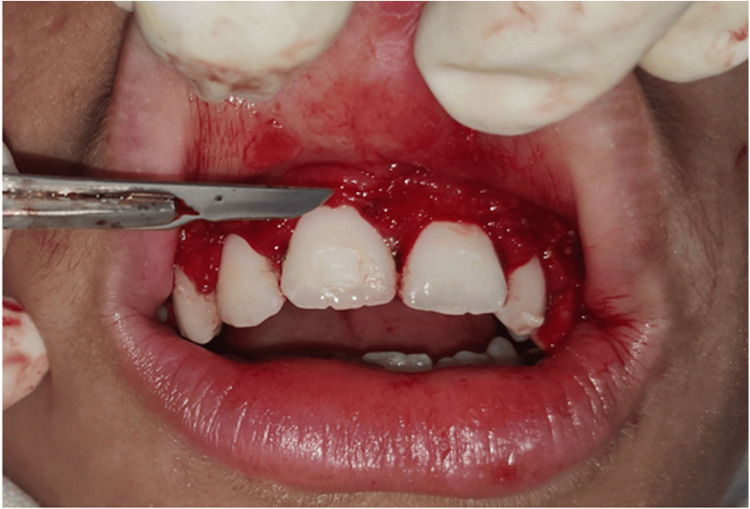
External bevel incision made at a 45-degree angulation

**Figure 4:**
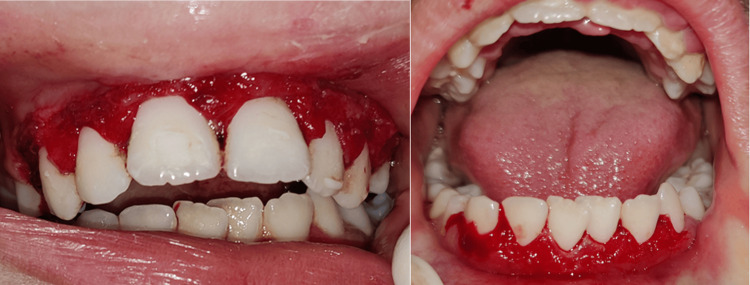
Postoperative photograph taken after gingivectomy and gingivoplasty, followed by oral hygiene prophylaxis

**Figure 5:**
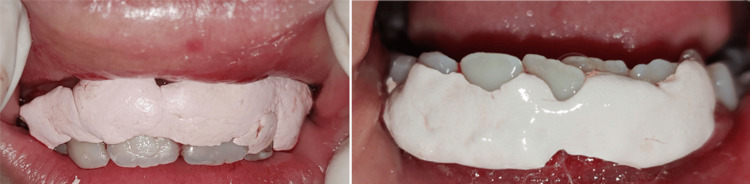
Perio-pack was placed to facilitate healing

The procedure started with local anesthesia administration. The depth of the pocket was marked with a pocket marker, and bleeding points were obtained (Figure 2). Gingivectomy was carried out using the conventional scalpel method with a number 15 BP blade. An external bevel incision at 45-degree angulation was done (Figure 3). Tissue tags were removed with a curette and scissors. Gingivoplasty was then carried out using a scalpel to achieve a smooth gingival surface (Figure 4). The excised tissue measuring 1.5×1.2 cm was sent for histopathological examination. This was followed by oral prophylaxis and the application of sodium fluoride gel to all tooth surfaces as part of preventive therapy. The patient was instructed to maintain strict oral hygiene. Bleeding was controlled using Chromostat and adrenaline, and Coe-Pack was subsequently placed to facilitate healing (Figure 5).

**Figure 6:**
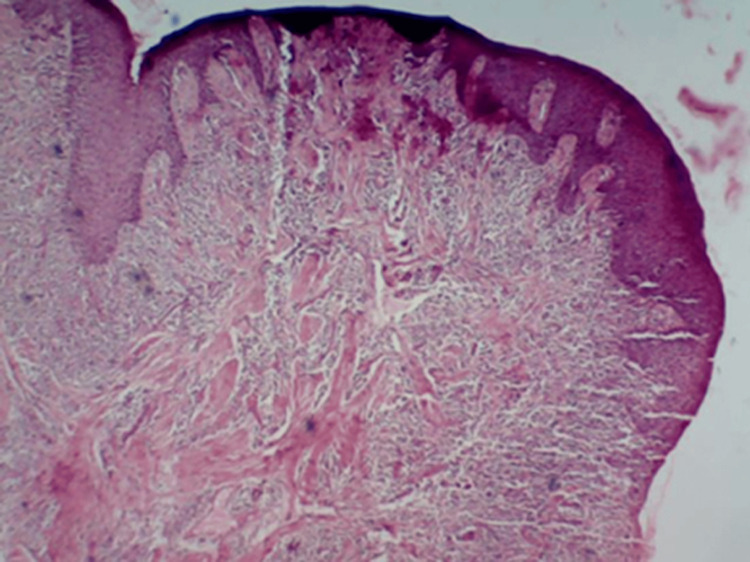
Histologic slide showing features of inflammatory fibrous hyperplasia

**Figure 7:**
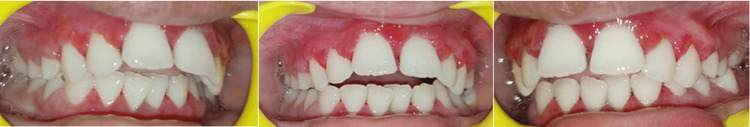
Intraoral photograph showing satisfactory healing two months postoperatively

Biopsy specimen showing parakeratinized stratified squamous epithelium. The epithelium was hyperplastic, with proliferating rete ridges exhibiting pseudoepitheliomatous hyperplasia. The underlying connective tissue consisted of dense collagen bundles and a dense mixed inflammatory cell infiltrate composed of neutrophils, plasma cells, and lymphocytes (Figure 6). These features were suggestive of inflammatory fibrous hyperplasia, which is seen in drug-induced gingival enlargement. Analgesics and chlorhexidine mouth rinse were prescribed, and the patient was advised to maintain strict oral hygiene. Seven days later, the patient returned for the removal of the periodontal dressing. The achieved gingival contour was found to eliminate pockets, promote satisfactory healing, and enhance aesthetic appearance. A follow-up examination conducted after two months showed satisfactory gingival contour with no evidence of periodontal pockets (Figure 7).

## Discussion

Drug-induced gingival enlargement occurs when drugs interfere with the metabolism of gingival fibroblasts, resulting in increased extracellular matrix deposition and tissue growth. This condition is often characterized by firm, fibrotic gingival tissue that covers the teeth, posing aesthetic and functional challenges such as difficulty in maintaining oral hygiene and a higher risk of periodontal infections [5].

A new clinical index for grading gingival enlargement includes Grade 0 (no signs of gingival enlargement), Grade I (enlargement confined to the interdental papilla), Grade II (enlargement involving the interdental papilla and marginal gingiva), and Grade III (enlargement covering three-quarters or more of the crown) [6]. The present case exhibited Grade III gingival enlargement.

The clinical manifestation of gingival enlargement usually develops within one to three months of starting treatment with the associated drugs. Gingival overgrowth often begins at the interdental papillae and is more common in the anterior segment of the labial surfaces. Gingival lobulations develop gradually and can be inflammatory or fibrotic, depending on the level of inflammation caused by local factors. The gingival overgrowth caused by these drugs is not only aesthetically displeasing but also hinders nutrition and access to oral hygiene, resulting in increased susceptibility to infection, caries, and periodontal problems [3,7]. The Turesky modification of the Quigley-Hein Index (TQHI) was used to evaluate supragingival plaque, and the patient had a score of Grade 3 [8].

The risk factors and causative agents that increase the abnormal growth of gingiva include age, genetic factors, drug doses, plaque inflammation, and bacteria [3,9]. For drug-influenced gingival conditions, plaque bacteria in conjunction with the drug are necessary to produce a gingival response [2].

Some authors report plaque elimination as a primary preventive measure for drug-induced gingival overgrowth, as there is a strong correlation between an increased plaque index and the severity of gingival overgrowth [10,11].

Phenytoin, carbamazepine, and sodium valproate are among the most commonly prescribed first-line drugs for the control of epilepsy [12,13]. Compared to children, older age groups have a higher plaque index, and therefore, are associated with more gingival overgrowth. Gingival overgrowth by phenytoin has the highest occurrence percentage ranging from 3% to 93% and shows a prevalence of 50% in patients on long-term drug therapy [14]. The prevalence data for the other antiepileptic medicines vary and are not uniform [15].

The documented cases of sodium valproate are minimal. The first case was reported in a 15-month-old child by Syrjänen and Syrjänen in 1979 [16], followed by a case in a 14-year-old girl by Behari in 1991, and in a nine-year-old girl by Anderson et al. in 1997. Another reported case involved a 20-month-old male child who developed gingival enlargement five months after initiation of therapy. The patient was under polytherapy with sodium valproate and clobazam [17]. In another reported case, gingival enlargement was observed in a five-year-old child who presented with difficulties in maintaining oral hygiene and speech, along with delayed cognitive skills [18]. Tan et al. in 2004 showed that the duration of the drug had a significant effect on gingival enlargement but not on the gingival index, plaque index, and probing pocket depth [19]. A case series documented by Maislla et al. in 2024 reported that children with microcephaly who received long-term antiepileptic therapy for two years exhibited oral manifestations, including gingival overgrowth and difficulty in maintaining oral hygiene. Among the cases reported, one child underwent polytherapy with sodium valproate and carbamazepine [20].

Hence, this case report aligns with the studies by Tan et al. (2004) and Maislla et al. (2024), which reported that a long-term duration of antiepileptic drug use (two years), combined with increased gingival and plaque indices, contributed to the development of gingival overgrowth. Due to the presence of significant generalized gingival enlargement, gingivectomy followed by oral prophylaxis was performed. This case is rare, as few instances of sodium valproate-induced gingival enlargement have been documented, and surgical management in a pediatric patient has not yet been reported.

## Conclusions

This case report documents a surgical intervention for sodium valproate-induced gingival overgrowth, aimed at improving the patient’s aesthetic profile as well as gingival and plaque indices. As long as the patient continues sodium valproate therapy, gingival overgrowth may persist; however, strict maintenance of oral hygiene can significantly reduce the likelihood of recurrence.
